# Statistics and Measurements

**DOI:** 10.6028/jres.106.010

**Published:** 2001-02-01

**Authors:** M. Carroll Croarkin

**Affiliations:** National Institute of Standards and Technology, Gaithersburg, MD 20899-8980

**Keywords:** calibration and measurement assurance, error analysis and uncertainty, design of experiments, history of NBS, interlaboratory testing, measurement methods, standard reference materials, statistical computing, uncertainty analysis

## Abstract

For more than 50 years, the Statistical Engineering Division (SED) has been instrumental in the success of a broad spectrum of metrology projects at NBS/NIST. This paper highlights fundamental contributions of NBS/NIST statisticians to statistics and to measurement science and technology. Published methods developed by SED staff, especially during the early years, endure as cornerstones of statistics not only in metrology and standards applications, but as data-analytic resources used across all disciplines. The history of statistics at NBS/NIST began with the formation of what is now the SED. Examples from the first five decades of the SED illustrate the critical role of the division in the successful resolution of a few of the highly visible, and sometimes controversial, statistical studies of national importance. A review of the history of major early publications of the division on statistical methods, design of experiments, and error analysis and uncertainty is followed by a survey of several thematic areas. The accompanying examples illustrate the importance of SED in the history of statistics, measurements and standards: calibration and measurement assurance, interlaboratory tests, development of measurement methods, Standard Reference Materials, statistical computing, and dissemination of measurement technology. A brief look forward sketches the expanding opportunity and demand for SED statisticians created by current trends in research and development at NIST.

## 1. Introduction

For more than 50 years, Statistical Engineering Division (SED) staff have played a critical role in the success of a broad spectrum of metrology projects at NBS/NIST. During this time, statistics at NBS/NIST has progressed with the constant goal of improving and characterizing measurement methods. Methods and publications which were developed early in the life of the division are still cornerstones for statistical analyses and are applied across all disciplines and metrologies. Over the years, existing methods have been refined and expanded and new methods have been developed to address recent challenges and take advantage of the statistical literature and the tremendous surge in statistical computing capability.

SED research contributions cover: quantification of uncertainty in measurements, statistical design of experimental investigations, monte carlo modeling, parameter estimation, stochastic modeling, exploratory data analysis and empirical modeling, model validation, computer intensive statistical methods, reliability analysis, statistical signal processing, image analysis, time series analysis, hypothesis testing, and quality control.

Statisticians participate in the planning of experimental studies and conduct rigorous uncertainty analysis of results and develop theoretical models to augment experimental work done by NIST collaborators. Examples of such work include Monte Carlo simulation of physical processes, such as neutron scattering, and stochastic differential modeling of aerosol particle spectrometers. Typically, SED staff develop long term relationships with collaborators in the other NIST laboratories and develop intimate knowledge of the scientific field in which they work. Here we highlight areas where SED contributes to metrology work at NIST with examples from recent collaborations along with an historical perspective for viewing statistical contributions to metrology.

## 2. History

### 2.1 Early Days

**Fig. 1:**
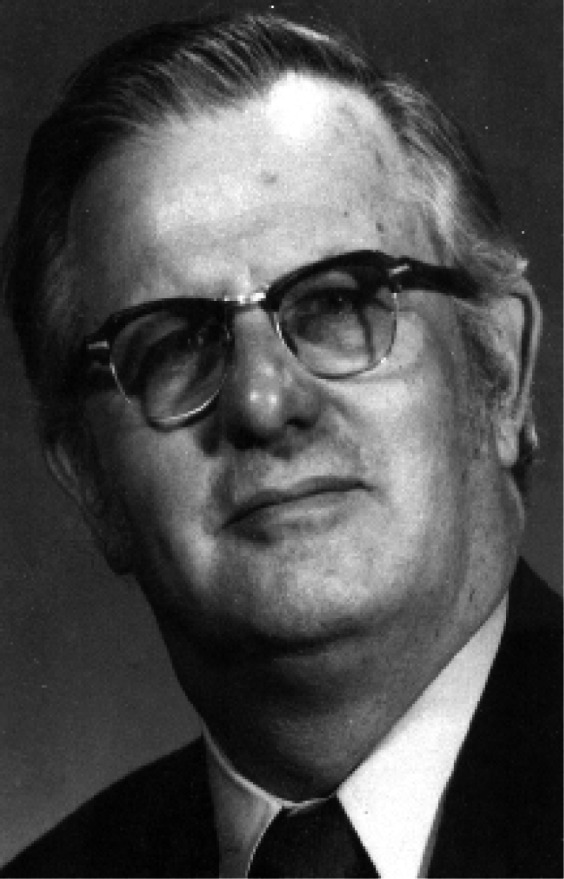
Churchill Eisenhart.

Churchill Eisenhart (see Fig. 1) came to NBS from the University of Wisconsin in 1946 when Edward Condon, Director of NBS, resolved to establish a statistical consulting group to “substitute sound mathematical analysis for costly experimentation” [26]. As the first Chief of the Statistical Engineering Laboratory (SEL), he shaped the direction that statistics would take at NBS for many years and personally laid the foundation for error analysis related to measurement science.

In its early days SEL, in its work with scientists at NBS, was drawn into several interesting activities as the Secretary of Commerce encouraged NBS to become involved in outside activities. The most important of these was the controversy over battery additive AD-X2 [13]. The NBS Director, A. V. Astin, had been pressured by various senators and the additive producer to test the additive for its ability to improve battery performance. The statisticians, under severe time constraints, were responsible for recommending experimental designs for testing the additive.

**Fig. 2:**
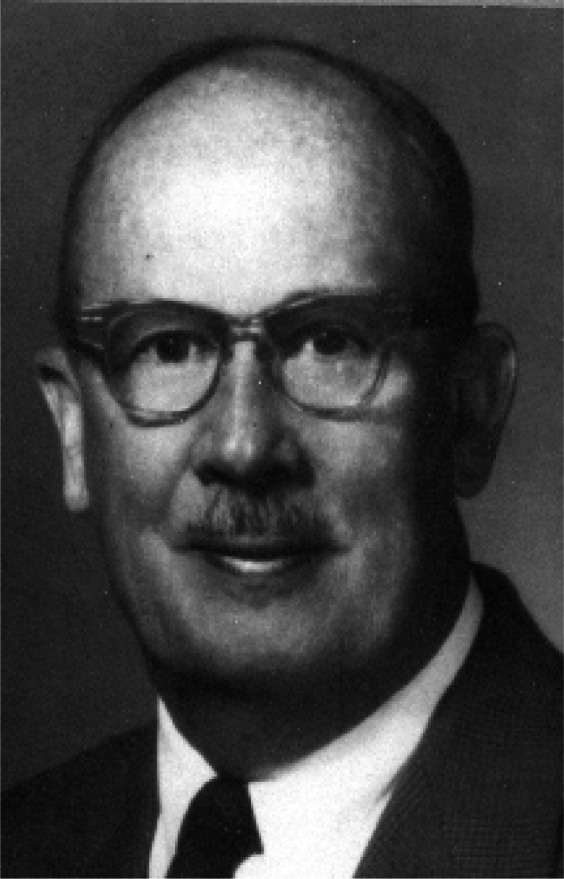
Jack Youden.

There were 32 batteries available for the test, and the manufacturer wanted to put all 16 batteries that were to be treated with AD-X2 on one charging line. The statisticians disagreed with the manufacturer and Jack Youden (see Fig. 2) proposed a design with the 32 batteries grouped in pairs for testing on three charging lines. On lines 1 and 2, both batteries of a pair were to be treated with AD-X2 or both were to be untreated. On line 3, there was one treated and one untreated battery in each pair. The statisticians were satisfied that this design for testing the electrical performance of the batteries could differentiate effects caused by AD-X2 from effects due to the charging line. They also insisted on a formal randomization scheme for selecting the batteries for treatment in order to avoid conflicts in the analysis. After this accomplishment, there ensued a brief moment of panic when they realized that a design was also needed for a visual test where the electrical plates would be disassembled and 45 paired comparisons would be made of treated and untreated batteries. Fortunately, Joseph Cameron found a suitable incomplete block design, thus avoiding the risk of having to construct such a design in the urgency of the moment [26].

The resulting analysis by SEL of this experiment, conducted by the Electrochemistry Section, confirmed that the additive had no significant positive effect on batteries, but in what was to quickly become an interesting sidelight of history, the Assistant Secretary of Commerce for Domestic Affairs announced that Astin had not considered the “play of the marketplace” in his judgment and relieved him as Director of NBS. Eventually, the National Academy of Sciences was called in to review NBS’s work, which was labeled first rate, and Astin was reinstated [24].

### 2.2 High Visibility Studies

NIST continues to rely on the Statistical Engineering Division for advice on experimental design, analysis, and interpretation whenever the institution is called upon as arbiter in technical conflicts of national importance. In 1970, when the Selective Service System was roundly and properly criticized for allowing birthdate to bias the draft lottery, Joan Rosenblatt, Chief of SED, led the NBS team that revised the procedures to ensure a fair and random lottery [1]. In the 1980s, when Congress was lobbied by the audio recording industry for protective legislation to require Digital Audio Tape systems to be fitted with a copy prevention decoder, NIST was asked to test the industry’s claim that a “notch” built into the recorded materials at high frequencies would not alter the quality of the recordings. A series of double-blind listening tests, designed by Keith Eberhardt, uncovered significant, although subtle, differences among subjects’ abilities to detect a notch and resulted in the legislation being denied [9].

In the 1990s, the Fire-Safe Cigarette Act was enacted to determine the practicability of developing a performance standard for less fire-prone cigarettes. The act was established to address the number one cause of fire deaths in this country, namely, cigarette ignition of upholstered furniture and bedding. Extensive testing by federal, private, and industrial laboratories of lit cigarettes on furniture mock-ups resulted in heated discussions as might be expected when an industry is facing potential regulation. NIST was asked to intervene, and Eberhardt led in the evaluation of screening tests conducted by the Fire Science Division of the Building and Fire Research Laboratory (BFRL). This work led to the development of two test methods and a carefully-designed interlaboratory evaluation of the test methods. Because standard statistical procedures for analyzing interlaboratory studies do not apply to the analysis of proportions, a methodology based on a simple model for “extra-binomial variation” [25] was developed specifically for analyzing this data. The cigarette industry responded with a new study that seemed to imply that fabrics selected by NIST for the study were atypical. Careful re-analysis of this data by Eberhardt demonstrated to a Technical Advisory Group made up of representatives from government, industry, consumer advocates, and testing laboratories the flaws in the industry’s analysis. SED collaboration with BFRL on cigarette ignition continues as the industry tries to respond to Congressional mandates for less fire-prone cigarettes and interlaboratory tests are mounted to assess progress in this direction.

### 2.3 Publications on Statistical Methods

One of the first large-scale contributions of the SEL to measurement science at NBS was the publication of NBS Handbook 91 [23] which has guided researchers at NIST for four decades in the planning and analysis of scientific experiments. In 1954, Eisenhart was approached by the Army’s Office of Ordnance Research and asked to produce a Manual of Experimental Statistics for Ordnance Engineers as a guide for military and civilian personnel with responsibility for planning and analysis of tests of Army equipment. Eisenhart assigned primary authorship to Mary Natrella, who had come to SEL from the U.S. Navy’s Bureau of Ships with extensive experience as a sampling inspection expert. The material was first printed for limited distribution as a series of five U.S. Army Ordnance Pamphlets as part of the AMC Engineering Design Handbook series.

Although Natrella was principal author, the material, which was several years in preparation, was the result of the combined experience and knowledge of the entire SED staff at the time. It proved to be of great benefit because of the clear elucidation of difficult statistical concepts accompanied by worked examples.

In 1963, the materials were published as NBS Handbook 91 and offered for sale to the general public. The 23 chapter headings listed in Table 1 (abbreviated) indicate the range of statistical methodologies that were employed in the NBS laboratories at the time. Allen V. Astin, Director of NBS at the time, says in the preface that, “although originally developed with the needs of the Army in mind, it promises to be equally useful to other groups concerned with research and development, both within and outside the Government.” Its strength came from the clarity of exposition, which was a hallmark of Natrella’s writing style, and from its detailed guidance, complete with numerical examples, on statistical computations that accompanied each test and procedure. In 1983, it was reprinted for commercial sale by Wiley Interscience as part of its Selected Government Publications series. In 1985, the American Society for Metals (ASM) published a condensation of four chapters on planning and analysis of comparative experiments as part of the Statistics Section of Volume 8 of the 9th edition of the ASM Handbook. Over the years, it has been NIST’s second-best selling publication.

A few years later, a compendium of papers by the Statistical Engineering Laboratory, was published in the NBS series on Precision Measurement and Calibration [19]. This book contains many historical papers including some of the papers referenced in this article and some that could not be referenced because of space considerations. The primary focus is error analysis of calibration and interlaboratory studies with the materials organized under the following topics:

- The Measurement Process, Precision, Systematic Error, and Accuracy
- Design of Experiments in Calibration
- Interlaboratory Tests
- Functional Relationships
- Statistical Treatment of Measurement Data
- Miscellaneous Topics

### 2.4 Design of Experiments

Statistical research in the Statistical Engineering Laboratory in the 1950s, led by William Connor and Marvin Zelen, focused on the development of experimental designs which were published as part of the NBS Applied Mathematics Series (AMS). AMS 48, AMS 54, and AMS 49 are devoted to factorial designs with factors restricted to 2 and 3 levels; AMS 62 is devoted to cyclic designs; and AMS 63 is devoted to partially balanced block designs. The papers on factorial designs contain fractional designs or subsets of complete factorials which are “optimized” for estimating individual factors and interactions among factors. This class of designs is probably the most important class for assessing the effect of various factors on measurement processes.

The designs were created by SEL staff with the assistance of many dedicated summer students; designs that were published before 1960 were created without electronic computers. The publications were offered for sale by the Government Printing Office for as little as 40 cents per copy.

Factorial designs are such an important class of experimental design and have found so many applications at NBS/NIST that it is impossible to give a representative accounting of their usage. A study in the Electromagnetic Technology Division of the Electronics and Electrical Engineering Laboratory in Boulder illustrates the use of factorial designs for optimizing a measurement process [6]. This particular study examined eddy current probe sensitivity as a function of coil construction parameters. Eddy currents can be used for detecting cracks in metal, such as airplane wings, and are measured by changes in the probe’s electromagnetic field. The experimental arrangement was a fractional factorial with each factor at two levels. The primary goal of the study was to identify probe construction factors and interactions with the largest effect on detector sensitivity as the probe is moved from an unflawed region of the metal to a flawed region of the metal. The analysis of sensitivity took advantage of an optimization scheme for pinpointing exact settings (over all factors) for maximizing sensitivity and produced an empirical equation for predicting sensitivity based on the levels of the various factors.

### 2.5 Error Analysis and Uncertainty

Uncertainty analysis is one of the primary responsibilities of the NIST statistician who is involved in reporting measurement results. Uncertainty quantifies the quality of a measurement result. In the early 1950s, precision and accuracy were commonly used for characterizing the quality of measurement processes although there was little common agreement or understanding as to their meaning and consequences. Eisenhart was drawn to this issue as it related to calibrations, which he called refined measurement methods. As Chief of SEL, he set out to put the concepts of accuracy and precision on a solid statistical basis.

In a paper that was to become the foundation for error analysis at NBS [10], Eisenhart synthesized his own work and the writings of statistical theorists and practitioners, Walter Shewhart, Edwards Deming, Raymond Birge, and R. B. Murphy, into concepts of quality control that could be applied to measurement processes. Three basic concepts in the paper have been embraced and practiced by metrologists at NBS ever since: (1) a measurement process requires statistical control; (2) statistical control in the metrology context implies control of both reproducibility and repeatability; and (3) a measurement result requires an associated statement of uncertainty which includes any possible source of bias. His paper was followed by others which laid the foundation for future developments in uncertainty analysis at NBS. Particularly noteworthy is a short paper by H. H. Ku [18] on propagation of error which is easily the most enlightening paper ever written on the subject.

The statistical determination of uncertainty in metrology is often complex, requiring careful consideration of the magnitudes of multiple sources of variability. A measurement may depend on these sources in a nonlinear way. Evaluating the individual components of uncertainty can require the combination of multiple sources of data, taken on various quantities upon which the primary measurement depends, both at NIST and from multiple outside laboratories. One example which illustrates these points is an SED collaboration with the Semiconductor Division of the Electronics and Electrical Engineering Laboratory. This work involved the indirect measurement of the geometry of thin pure-copper films, using an approach which exploits the relationships among resistance, resistivity, and conductor geometry [29]. The uncertainty analysis for the proposed technique incorporates interlaboratory test data on resistance, as well as a detailed analysis of the nonlinear relationship between resistance and resistivity, as estimated from extensive historical data.

Research on the subject of uncertainty is still evolving, and recent work takes advantage of modern statistical techniques such as Bayesian methods which provide a unified approach to combining relevant information in the measurement experiment [20].

## 3. Calibration and Measurement Assurance

Calibration is the assignment of a value to a test item or an instrument based on measurements made on the test item and on a reference standard with known value. Calibrations are of two types: (1) single-point calibration such as assignment of a mass to an unknown weight and (2) calibration over a regime such as a calibration of a linewidth standard from 0.1 µm to 10 µm. Experimental configurations, called calibration designs, for single-point calibrations specify measurements to be made on test items and reference standards. Designs of this type are the foundation for artifact calibrations at NIST. The solutions to these designs are based on restrained least-squares techniques [34] where the known value of the reference standard(s) is the restraint on the system of equations.

The Statistical Engineering Division has created a large portfolio of designs for NIST calibration laboratories and adds new designs to this collection to respond to specific situations, as needed, or to take advantage of advances in instrumentation and metrology. Recently, an automated balance that was introduced into the NIST mass laboratory required new designs to take advantage of the high precision of the balance and also deal with the limitations that it imposed on the experimental setup.

The contributions of statisticians at NIST to calibration designs date from the late 1950s when Joseph Cameron seized on advances in experiment design and electronic computing to introduce new calibration designs into NBS laboratories. The earliest designs of this type were created for intercomparing mass standards or weights, and were referred to as “weighing designs”. Cameron and Charles Reeve created designs for a broad range of dimensional and electrical quantities that include: the length of a gage block, roundness of a sphere, mass of a weight, degree of an angle block, voltage of a standard cell, resistance of a one ohm resistor, and the like, which are the basis for calibrations at NIST and throughout the U.S. metrology community. The unique aspect of the designs created by the statisticians is that they each have provision for a check standard to be “calibrated” with the test artifacts.

The check standard database is the basis for applying statistical control theory to measurement processes, and the statisticians worked to implement these strategies in the calibration laboratories of NBS. They also merged the check standard concept and quality control procedures to form a cohesive practice, known as measurement assurance, as a means of tying measurement results to a specified reference and quantifying uncertainty relative to the reference base. The first documentation of a measurement assurance program in a NBS calibration laboratory appears to be a tutorial on mass calibrations [27]. Measurement assurance programs now abound in metrology areas as diverse as dimensional measurements and semiconductor devices, and statistical control procedures, based on check standards, are the basis for controlling the output of NIST calibration processes.

Of equal importance, measurements on check standards form the basis for uncertainty determinations in many areas of metrology. In collaboration with scientists in the calibration laboratories, statisticians develop error models, for explaining sources of variability in the measurement process that are applicable to measurements on the check standards. This is critical to the assessment of uncertainty because measurements on check standards are the only recurring measurements in a calibration setting; thus they provide the only data for estimating long-term components of uncertainty which can be related to the uncertainties of values assigned to test items [7].

As mentioned previously, NIST also provides calibrations of quantities, such as force, where an instrument, such as a force sensor, is calibrated over a region of interest. The resulting function is a calibration curve which defines the relationship between the surrogate measurement and its reference. In general, neither the calibration curve nor its functional form is known and must be estimated from experimental data. Strategies for estimating the calibration curves under various scenarios are continually explored by SED statisticians. The difficult statistical task of computing the uncertainty of the “calibrated value” from the inverse of the calibration curve is also the domain of the SED. Eisenhart solved the problem of the uncertainty of the calibrated value for a single application of a linear calibration curve [44] in 1935. However, the general solution for multiple applications of a calibration curve has been an open problem in the metrology community for years; a solution using a tolerance interval approach [22] is the result of extensive experience with NIST calibration activities.

Because the list of papers on calibration and measurement assurance is too extensive for this publication, the reader is advised to go to http://www.itl.nist.gov/div898/pubs/slist.html for specific publications and to http://www.nist.gov/stat.handbook/mpc/section3/mpc34.htm for a catalog of calibration designs for weights, standard cells for voltage, resistors, gage blocks, angle blocks, roundness standards, and humidity standards.

## 4. Interlaboratory Tests

One of the most widely used tools for characterizing and validating measurement methods is the interlaboratory test where test results are gathered on the same or equivalent materials by several qualified laboratories. Statisticians are involved in the design and analysis of interlaboratory tests over a huge range of disciplines at NIST. Statisticians are members of teams that design the initial interlaboratory experiments, providing both analysis, interpretations and recommendations for further intercomparisons. The analysis tools depend not only upon the problem at hand but also on the purpose of the interlaboratory test and are not limited to specific statistical techniques, a popular misconception.

Interlaboratory tests sponsored by NIST are often undertaken for the express purpose of producing a consensus value. In such a case, the goal drives both the design of the experiment and the analysis which must frequently deal with the problem of outlying laboratories. As in all collaborations, but to a greater degree for important international comparisons, the statistician must not only recommend and implement statistical methods that are appropriate to the task but must also win the confidence of the participants. The solution is never his or her choice alone, and much care is taken to ensure that all parties understand and concur with the method of analysis. A study to determine a calibration factor for relating infrared absorption measurements to the interstitial oxygen content of silicon is an example. NIST statisticians were responsible for planning the experiments and estimating the final conversion factor and associated uncertainty for a wide range of oxygen contents from round robins of both infrared and absolute measurements [2]. This was an important study for the world-wide semiconductor industry as many measurements are now slaved to the calibration factor. Problems that had to be dealt with included non-equivalence among samples that were circulated to the participants and suspect results from some absolute measurements.

In 1990, the International Temperature Scale (ITS-90) replaced the 1968 International Practical Temperature Scale (IPTS-68). Because of a discontinuity in the 1968 scale that led to anomalies in temperature differences between the two scales in the range 630 °C to 1064 °C, the Consultative Committee on Thermometry of the International Committee for Weights and Measures, through its Working Group 2, organized a collaborative effort among national metrology institutes (NMIs) to generate new experimental data for type S thermocouples in that range. The NIST statisticians were responsible for creating new reference functions and inverse functions for type S thermocouples [3, 4]. These functions are now the basis for all temperature measurements within the range of these thermocouples. To mitigate the effect of outlying laboratories, the reference equation and associated uncertainties were computed using iteratively reweighted least squares regression.

Improvements in the design and analysis of interlaboratory tests in SED began in the 1960s when W. J. Youden sought to shed light on errors in measurement processes through experimental design. In his work with chemists and ASTM committees, Youden left a huge body of literature on the subject. He approached interlaboratory testing as a means of uncovering biases in measurement processes, and the so-called Youden plot [33] has become an accepted design and analysis technique throughout the world for comparing precision and bias among laboratories. Work in graphical methods, which began with the Youden plot, continues today, notably in recent work of NIST chemist David Duewer [8].

Likelihood and non-parametric methods were pioneered by John Mandel, culminating in a book on the analysis of two way tables [21]. Mandel, although not a staff member of the SEL, spent his career as a statistician working within the chemical community of NBS and with the ASTM community to develop methods for quantifying within-laboratory and between-laboratory precision. His methodology, originally applied to the chemical and paper industries, has been codified in national [1] and international [17] standards. New interpretations of some of Mandel’s work by SED statisticians [53], and the solution of outstanding problems, notably estimation where not all laboratories are operating with the same precision, has garnered recognition within the statistical community.

Recently, the statistical modeling of interlaboratory test data has led to advances in the theory of linear mixed-effects models from graphical and likelihood approaches and to Bayesian solutions to combining measurements over multiple laboratories or methods [32].

International comparisons of basic metrological standards are currently an important component of SED activities at NIST. Studies, known as key comparisons, for comparing measurements among NMIs have taken a critical place in the NIST mission. Their purpose is to establish the degree of equivalence of national measurement standards maintained by NMIs and provide for the mutual recognition of calibration and measurement certificates. SED staff are contributing to key comparisons for measurements of temperature, thermal conductivity, differential and absolute pressure, humidity, vibration and acceleration, and basic electrical quantities, including capacitance, sound, ultrasonic power, and linescale.

Key comparisons serve as the technical basis for judging measurements around the world and must, therefore, accurately reflect the true relationships between measurement systems maintained by NMIs. SED statisticians provide guidance on comparison designs to ensure that data collection will be as effective as possible for quantifying both differences and uncertainty and implement analyses which account for covariances in the measurements and ensure that uncertainties have a specified confidence level.

## 5. Development of Measurement Methods

Development of new measurement methodology is probably the most critical element in the NIST mission. SED statisticians contribute to these efforts via collaborative research efforts which typically proceed in several stages. Initially, the problem is studied for proper understanding, and statistical issues are identified and communicated to the research team. An experiment is designed and statistical methods are applied to the resulting data. New statistical methods, or modifications of existing methods, are often required. Finally, statisticians participate in the preparation of NIST written records or archival journal publications. Some collaborations are one-time associations; others proceed iteratively over several years, with the results of one project providing the foundation for the next investigation.

A collaboration on magnetic trapping of ultra cold neutrons is currently underway with the Ionizing Radiation Division of the Physics Laboratory [15]. SED staff are part of an international team of researchers from NIST, Harvard University, Los Alamos National Laboratory, and the Hahn-Meitner-Institute in Berlin. The team proposed a new technique to trap ultra cold neutrons in a magnetic field. With this technology, the team plans to make a high precision measurement of the mean lifetime of the neutron. Along with other experimental data, the mean lifetime of the neutron allows one to test the consistency of the standard model of electroweak interactions. The mean lifetime of the neutron is also an important parameter in astrophysical theories. Eventually, this method should yield a lifetime estimate with an uncertainty 10 to 100 times smaller than the current uncertainty.

**Fig. 3:**
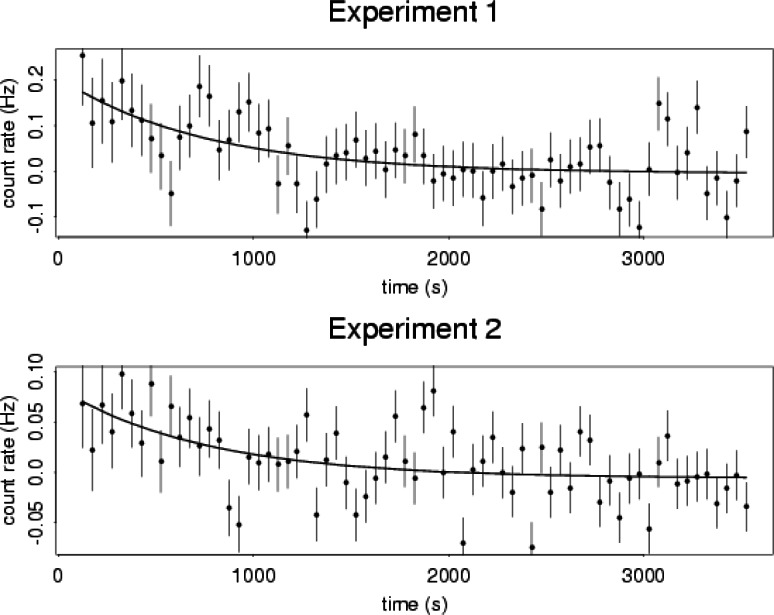
These plots show observed data with associated standard uncertainties for two experiments. For each experiment, the predicted count rate is shown as a solid line.

**Fig. 4:**
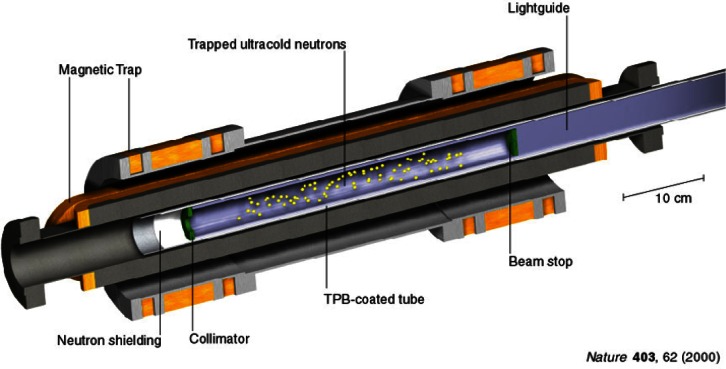
Schematic diagram of the magnetic trap which confines ultra cold neutrons.

Statistical contributions to this project include planning of a multi-run experiment which is performed at the NIST Cold Neutron Research Facility. A magnetic trap is filled with neutrons for a prescribed time. After the filling stage of each run, the neutron beam is blocked and decay events plus background events are observed during the event observation stage of each run. Based on a birth-death stochastic model of the neutron trapping process, the statisticians have developed an algorithm which determines the optimal amount of time for filling and the optimal amount of time for observing events. This algorithm has played a critical role in the planning of the second generation of the experiment now underway. Some of the data from these experiments and a schematic diagram of the magnetic trap are shown in Figs. 3 and 4.

Another example is a collaboration which began in 1988 with the Optoelectronics Division of the Electronics and Electrical Engineering Laboratory to develop statistical signal processing methods for analysis of time-domain optoelectronic response measurements [12]. Optoelectronic devices are critical for high bandwidth measurements of high performance optical fiber systems. A photodiode converts an optical signal into an electrical signal. This electrical signal is detected with a high speed equivalent time sampling oscilloscope. Both the photodiode and oscilloscope have impulse response functions which distort the signal of interest.

SED staff are developing statistical methods and associated software for calibration of high-speed digital sampling oscilloscopes and characterizing the impulse response of photodiodes. Statistical tasks include development of estimation methods and algorithms for timebase distortion estimation and correction; drift estimation; signal alignment; and timing jitter estimation. SED staff have developed statistical methods and associated software used in a measurement system for sampling oscilloscopes (up to 50 GHz) to correct signals for systematic errors due to timebase distortion, drift, and jitter.

In the near future, the experimental work will be extended to higher wavelengths. Statistical methods will be developed to evaluate the overall uncertainty of the estimated power and phase spectrum of the oscilloscope and photodiode impulse response functions, and time domain measurements will be compared to heterodyne measurements.

## 6. Standard Reference Materials

One of the ongoing metrology activities that SED supports at NIST is the certification of Standard Reference Materials (SRMs). SRMs are artifacts or chemical compositions that are manufactured according to strict specifications and certified by NIST for one or more chemical or physical properties. SRMs are a primary vehicle for disseminating measurement technology to industry. In the 1970s, the Statistical Engineering Division entered a phase of intensive interactions with developers of SRMs at NIST. This activity persists to this day, and SED staff are heavily involved in the certification of large numbers of SRMs each year.

The largest number of SRMs are chemical compositions from the Analytical Chemistry Division of the Chemical Sciences and Technology Laboratory. These SRMs are incredibly varied and many, particularly those for environmental applications, are certified for the percentage concentration of 50 constituents or more where the constituents are contained in a natural matrix such as sludge from a river bottom. Typical multi-constituent SRM materials include marine sediment; uric acid; Lake Superior fish tissue; Portland cement and urban air particulate matter. Multi-constituent SRMs offer special challenges because the experimental configuration is often highly unbalanced and cannot always be treated by standard statistical methods.

SRMs from other NIST laboratories and measurement areas cover a variety of applications that include sinusoidal roughness of steel blocks; magnification level for scanning electron microscopes; resistivity of silicon wafers; transmittance of optical density filters; characterization of radionucleides in ocean sediment, Rockwell C Scale for hardness; fracture toughness of ceramics; wavelength reference for a hydrogen cyanide absorption cell; and thermal resistance of fibrous glass insulation. A listing of publications related to the certification of SRMs can be found at http://www.itl.nist.gov/div898/pubs/subject/srm.html.

The development of a new SRM typically takes 2 to 5 years and encompasses design of a prototype, stability testing, quantification of sources of error, and certification and uncertainty analysis. Statisticians collaborate with NIST chemists and scientists and advise on the design and analysis of experiments at all phases; develop estimation methods; reconcile interlaboratory differences; test and estimate the effect of inhomogeneity on the certified value; and combine all information to produce a certified value and statement of uncertainty. A method for combining measurements over multiple laboratories or methods which have significant differences is addressed in a 1991 paper [28].

**Fig. 5:**
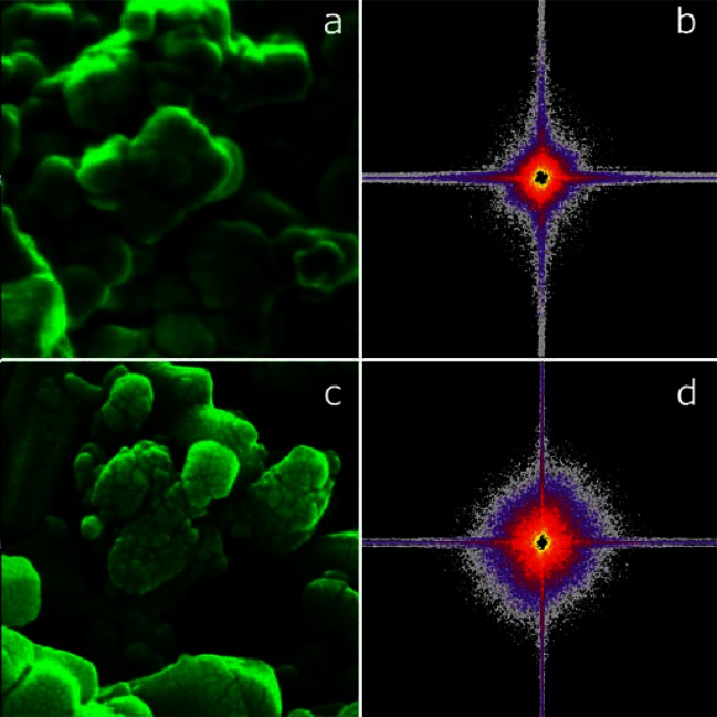
Parts (a) and (c) of the figure show two micrographs taken with an SEM. Micrograph (a) appears to be far less sharp than micrograph (c), taken when the same instrument was operating more optimally. Parts (b) and (d) show the 2-D spatial Fourier frequency magnitude distributions for the two micrographs. Notice that the magnitude distribution of the Fourier transform of the images is wider for (c) than for (a). Treating the normalized spectrums as probability density functions, the sharpness of an SEM image can then be determined numerically by its multivariate kurtosis.

Non-standard metrologies, such as video imaging, are also disseminated via SRMs and often present special challenges. Automation of semiconductor production requires scanning electron microscopes (SEMs) that are capable of measuring feature sizes without human intervention for long periods of time. An SED staff member has collaborated with the Precision Engineering Division of the Manufacturing Engineering Laboratory to develop a statistical method for testing the performance of scanning electron microscopes [35] that is the basis for a new SRM (see Fig. 5).

A simple explanation is that an SEM image with fine details is “sharp”. If the normalized spatial spectrum is treated as a probability density function, a sharp SEM image corresponds to a spectrum which has a large shoulder or a flat shape. The test procedure monitors the kurtosis (flatness) statistic to detect any increase in kurtosis that signals degradation in sharpness. This type of collaboration, which begins with an industrial measurement problem and results in artifacts and test methods that allow proper use of the artifacts, requires perhaps several years work to bring to fruition as the methodology must be developed, tested at NIST, and finally tested in the industrial setting.

## 7. Statistical Computing

The ubiquitous use of statistics at NIST has come about for many reasons, one of which is certainly the development of state-of-the-art statistical computing tools within SED. In the early 1960s, Joseph Hilsenrath of the Thermodynamics Section, Heat and Power Division, conceived the idea of a spreadsheet program for scientific calculations. Together with Joseph Cameron and the support of several NBS sections, this idea led to a program called Omnitab [5]. Omnitab is an interpretive computing system with a command structure in English that performs scientific calculations on data in columns in a worksheet.

When Cameron became Chief of SEL, he formed a team, headed by David Hogben, to complete the development of Omnitab as a sophisticated statistical package. By 1966, it was already strong in data manipulation, regression analysis with related diagnostic graphics and tests, one and two-way analysis of variance, special functions, and matrix operations. It quickly became the standard tool for statistical calculations at NIST. It was so innovative at the time that when Brian Joiner left SEL in the 1960s to teach at Pennsylvania State University, he took a copy of Omnitab with him for his students. A few years later, Joiner formed a company that revised the code and offered it for sale as the commercial package, Minitab.

Omnitab is strong on analytical procedures but not on graphics output. In 1969, when James Filliben brought his perspective on exploratory data analysis (EDA) to NBS, he immediately saw the need for software with strong graphics capability, and he set about developing code to support his consulting activities that incorporated the best features of EDA. There was never a steering committee for this project as there was for Omnitab, but from the breadth of problems and data encountered in the NBS laboratories, a diverse and versatile package, called Dataplot [14], was conceived. The package is a workhorse for graphical and statistical analysis at NIST and is a repository for datasets from important NIST experiments. Because it is a free and down-loadable resource maintained by the Information Technology Laboratory, Dataplot has recently been interfaced with an on-line statistics handbook that is under development within the Statistical Engineering Division and SEMATECH. From the handbook pages, the reader can run examples of statistical approaches presented in case studies in the handbook.

## 8. Dissemination of Measurement Technology

NIST, as a research and development body, is charged to disseminate its measurement technology to its client industries. There are several mechanisms for this delivery, and, of course, joint publication of papers and technical notes with NIST metrologists is one of the primary ways in which statisticians contribute to this effort. Another mechanism is via contributions to ASTM and ISO committees which develop and publish standards where valid statistical procedures and analysis are critical to the proper execution and/or validation of the measurement method. Staff contribute to ISO Technical Committee 69 on Statistical Methods on a continuing basis and provide support for drafting and review of documents in areas related to metrology. A document on Statistical Methods for Assessing Uncertainties in Scientific Laboratories that is in draft will provide statistical methods for designing metrology experiments and estimating components of variability that are needed to implement the ISO Guide [16] on uncertainty analysis.

A third mechanism for disseminating technology is training. During the 1990s, while Robert Lundegard was Chief of SED, he encouraged the development of a broad spectrum of training courses and workshops for NIST staff and their client groups. Workshops on Design of Experiments, Regression Analysis, Analysis of Variance, Statistical Intervals, and Time Series are an on-going effort of the division. Some of these workshops have been presented multiple times and continue to attract large audiences both from within and without NIST.

On the other hand, there are workshops dedicated to particular measurement technologies. For over 10 years, SED statisticians and staff from the Electricity Division of EEEL gave a 5 day Electrical Measurement Assurance Program workshop with emphasis on measurement techniques, error characterization, experiment design, and control of the measurement process. Nowadays statisticians contribute to an Advanced Mass Measurement Workshop, sponsored by the NIST Office of Weights and Measures, and to workshops on mass metrology and uncertainty analysis for NIST counterpart laboratories in Latin and South America that are given jointly and in cooperation with staff of the Precision Engineering Division of the Manufacturing Engineering Laboratory. These workshops provide background in theory, procedures, and statistics that are required for mass calibration at low levels of uncertainty. Workshops on certifying chemicals as reference materials and assessing related uncertainties are given at venues such as the American Chemical Society annual meetings.

Workshops on the broader subject of uncertainty analysis that cover a range of metrology applications are part of SED’s commitment to furthering the understanding of this topic; these workshops last a few hours or a few days and take place at conferences such as the International Laboratory Accreditation Conference (ILAC) and at the annual Measurement Science Conference.

Recently, the availability of the internet has led the Division to explore ways of using this resource to reach its client base of NIST scientists and NIST’s own clients in the most expeditious manner. Two projects which take advantage of dissemination over the World Wide Web are described below.

The first is a joint project with SEMATECH, a consortium of major U.S. semiconductor manufacturers. It began as an effort to update NBS Handbook 91 by providing modern statistical techniques and examples related to semiconductor measurements and evolved into the NIST/SEMATECH Internet Engineering Statistics Handbook [31] for the World Wide Web. The purpose of the publication is to extend the benefits of modern statistical design and analysis to the engineering and scientific communities and make the information more accessible to scientists in the NIST metrology laboratories.

Topics addressed in the eight chapters are as follows:

- Exploratory Data Analysis
- Measurement Process Characterization
- Production Process Characterization
- Process Modeling
- Process Improvement (design of experiments)
- Product and Process Monitoring and Control
- Product and Process Comparisons
- Product Reliability

The approach is problem-oriented and includes detailed case studies from the semiconductor industry and NIST laboratories that illustrate statistical approaches to solving engineering and scientific problems. The treatment of examples has evolved from reliance on detailed calculations with explicit formulas, as in Handbook 91, to analyses based on graphical and analytical output from statistical software packages.

Over the last few years, SED has been approached by scientists from both within and outside NIST for guidance on selecting statistical software for specific purposes. The Standard Reference Datasets (StRD) [30] project, which is a joint effort with the Mathematical and Computational Sciences Division of ITL and Standard Reference Data Program of Technology Services, is a response to that need. It addresses the numerical accuracy of statistical software. Because potentially serious numerical accuracy problems sometimes remain undetected despite extensive testing and continuing software development, an easily accessible repository of reference datasets has been constructed to help software developers and users assess the numerical accuracy of the statistical software.

The StRD Web pages provide datasets with certified values for assessing the accuracy of software for (1) univariate statistics, (2) analysis of variance, (3) linear regression, and (4) nonlinear regression.

The collection includes both generated and real-world data from the NIST laboratories and other sources of varying levels of difficulty. Generated datasets are designed to challenge specific computations. These include the classic datasets developed in SEL during the 1970s for testing linear regression algorithms.

These Web pages are attracting a great deal of attention. In a pair of recent articles [36, 37], Bruce McCullough surveyed statistical software packages from several vendors using the StRD datasets and compared results across packages. This has led to increased awareness by the software developers, some of whom cite McCullough’s article and the StRD pages to show that their results agree favorably with the certified values from NIST.

## 9. Future Trends in Statistics at NIST

The fact that international recognition of measurement services that the NMIs offer is directly supported by regular participation in relevant key comparisons suggests that this area will be an increasingly important source of activity for some time.

Other areas of expanding opportunity include information technology, biotechnology, and computational chemistry. These new research areas at NIST will call for increased competence in methodology for dealing with very large and complex data sets, such as arise in image and internet traffic data, for example.

There will be opportunity to leverage advances in statistical methodology and information technology for NIST applications. Recent progress in the field of statistics is making available new techniques in areas such as Bayesian methods, non-parametric analysis, generalized additive models, and tree-based modeling methods.

During the past decade, with increased computing power and new research developments, Bayesian statistical methods have become practical in diverse areas of statistical applications. Bayesian methods provide a framework for optimally combining information from multiple sources, resulting in simpler and improved statistical analyses. Despite the widespread growth in Bayesian methods, the field of metrology has not taken advantage of these methods. Both NIST researchers and their customers have much to gain from these methods. Recognizing the potential has encouraged NIST statisticians to begin the exploration of Bayesian methods in several metrological applications.

## Figures and Tables

**Table 1:** Table of Contents of *Handbook 91: Experimental Statistics* by Mary Natrella

Ch. 1. Some basic statistical concepts
Ch. 2. Characterizing measured performance
Ch. 3. Comparing with respect to the average
Ch. 4. Comparing with respect to variability
Ch. 5. Characterizing linear relationships
Ch. 6. Polynomial and multivariable relationships
Ch. 7. Characterizing qualitative performance
Ch. 8. Comparing with respect to a two fold classification
Ch. 9. Comparison with respect to several categories
Ch. 10. Sensitivity testing
Ch. 11. Considerations in planning experiments
Ch. 12. Factorial experiments
Ch. 13. Randomized blocks, Latin squares
Ch. 14. Experiments to determine optimum conditions
Ch. 15. Some shortcut tests for small samples
Ch. 16. Tests which are independent of distribution
Ch. 17. The treatment of outliers
Ch. 18. Control charts in experimental work
Ch. 19. Extreme-value data
Ch. 20. The use of transformations
Ch. 21. Confidence intervals and tests of significance
Ch. 22. Notes on statistical computations
Ch. 23. Expression of uncertainties of final results
